# Genome-Wide Association Study for Resistance to *Phytophthora sojae* in Soybean [*Glycine max* (L.) Merr.]

**DOI:** 10.3390/plants13243501

**Published:** 2024-12-15

**Authors:** Hee Jin You, Ruihua Zhao, Yu-Mi Choi, In-Jeong Kang, Sungwoo Lee

**Affiliations:** 1 Department of Crop Science, College of Agriculture and Life Sciences, Chungnam National University, Daejeon 34134, Republic of Korea; heejinyou0410@gmail.com (H.J.Y.);; 2 National Agrobiodiversity Center, National Institute of Agricultural Sciences, Rural Development Administration, Jeonju 54874, Republic of Korea; 3 Division of Crop Cultivation and Environment Research, Department of Central Area Crop Science, National Institute of Crop Science, Suwon 16613, Republic of Korea

**Keywords:** soybean, genome-wide association study (GWAS), resistance to *Phytophthora sojae*, linkage disequilibrium (LD) block, haplotype, multi-locus model

## Abstract

*Phytophthora sojae* (Kauffman and Gerdemann) is an oomycete pathogen that threatens soybean (*Glycine max* L.) production worldwide. The development of soybean cultivars with resistance to this pathogen is of paramount importance for the sustainable management of the disease. The objective of this study was to identify genomic regions associated with resistance to *P. sojae* isolate 40468 through genome-wide association analyses of 983 soybean germplasms. To elucidate the genetic basis of resistance, three statistical models were employed: the compressed mixed linear model (CMLM), Bayesian-information and linkage disequilibrium iteratively nested keyway (BLINK), and fixed and random model circulating probability unification (FarmCPU). The three models consistently identified a genomic region (3.8–5.3 Mbp) on chromosome 3, which has been previously identified as an *Rps* cluster. A total of 18 single nucleotide polymorphisms demonstrated high statistical significance across all three models, which were distributed in eight linkage disequilibrium (LD) blocks within the aforementioned interval. Of the eight, LD3-2 exhibited the discernible segregation of phenotypic reactions by haplotype. Specifically, over 93% of accessions with haplotypes LD3-2-F or LD3-2-G displayed resistance, whereas over 91% with LD3-2-A, LD3-2-C, or LD3-2-D exhibited susceptibility. Furthermore, the BLINK and FarmCPU models identified new genomic variations significantly associated with the resistance on several other chromosomes, indicating that the resistance observed in this panel was due to the presence of different alleles of multiple *Rps* genes. These findings underscore the necessity for robust statistical models to accurately detect true marker–trait associations and provide valuable insights into soybean genetics and breeding.

## 1. Introduction

Soybean [*Glycine max* (L.) Merr.] is the most economically important crop among legumes, and its annual production and cultivated area worldwide have doubled from 161 to 353 million metric tons and from 74 to 126 million hectares, respectively, from 2000 to 2020 [1]. Almost 40% of the edible vegetable oil consumed worldwide is produced from soybeans [2]. Soybean crops suffer from various pests and pathogens, which are the main factors decreasing their yield and quality [3,4]. Of these, Phytophthora root rot (PRR), caused by *Phytophthora sojae* (Kaufmann and Gerdemann), is one of the most destructive diseases of soybeans worldwide. PRR can occur throughout soybean growth and cause damping-off in seedlings as well as root and stem rot in mature plants, resulting in yield losses [5]. 

The pathogen enters soybean plants through their roots, leading to symptoms such as damping-off, root rot, and wilting. Its destructive nature is exacerbated by the formation of thick-walled oospores that can survive in soil for extended periods. Under favorable conditions, oospores germinate, giving rise to sporangia, which, in turn, produce motile zoospores. These zoospores swim through soil water to reach and infect the roots of susceptible plants. Upon encysting the root surface and penetrating the appressoria, the pathogen establishes an infection in the root tissue. The pathogen causes symptoms as the mycelium grows. This pathogen spreads through waterborne spores, making it particularly challenging to control. Cultural practices, including proper drainage and rotation with non-host crops, are often employed to manage PRR, and fungicides may offer additional control measures. The most desirable way to control PRR is to grow soybean cultivars resistant to *P. sojae* [6].

Genetic host resistance, controlled by single resistance genes, is the primary management method for PRR. To mitigate the impact of *P. sojae*, farmers often rely on soybean varieties, employing *Rps* (resistance to *Phytophthora sojae*) genes. Since the identification of the first resistance gene to *P. sojae* in the 1950s [7], more than 30 *Rps* genes/alleles have been identified and mapped to ten soybean chromosomes (chrs.). Many *Rps* genes/alleles are mapped to chrs. 3, 13, and 18 [8,9]. These studies provide a foundation for developing soybean varieties with *P. sojae* resistance, and some *Rps* genes such as *Rps1a*, *Rps1b*, *Rps1c*, and *Rps1k*, have been deployed in commercial soybean varieties in Canada and the U.S. [6]. However, the pathogen has a remarkable ability to overcome resistance mechanisms, and the intensive use of a few specific *Rps* genes in commercial cultivars, leads to the shortened longevity of *Rps* genes [6]. Consequently, *Rps1a*, *Rps1c*, and *Rps1k* are no longer effective in the U.S., though they are highly utilized in Brazilian soybean breeding programs [10,11].

The efficacy of an *Rps* gene is generally reliable for only 8–15 years, and these genes can be overcome by emerging pathotypes of *P*. *sojae* isolates in the field [12]. A recent 10-year interval investigation revealed that the diversity of pathotypes was altered and varied among states in the U.S. Thus, selecting and deploying appropriate *Rps* genes based on the locally dominant pathotypes of *P. sojae* populations, is critical for the efficient management of PRR. This emphasizes the need for ongoing research on developing new resistant varieties. From a long-term perspective, the continuous discovery of valuable resistant germplasms and the identification of novel resistance genes are critical to cope with the dynamic evolution of *P. sojae* populations over time. Genetic diversity based on diverse soybean germplasm resources plays a pivotal role in exploring the valuable traits of interest such as yield, agronomic traits, the resistance to abiotic and biotic stresses, and seed quality-related traits [13,14,15]. As valuable genetic resources, large soybean collections have been established and utilized in China, U.S., Japan, and Korea [16,17,18,19]. 

Genome-wide association studies (GWASs) using a panel of diverse germplasms have proven to be a promising approach for dissecting genomic regions significantly associated with target traits [20]. Although genetic mapping using biparental populations is a powerful approach for identifying genomic regions of traits of interest, it has limitations in terms of allelic diversity and genomic resolution. In contrast, a GWAS utilizes the genetic diversity of a panel of unrelated individuals to capture historical recombination by creating shorter linkage disequilibrium (LD) blocks, allowing the identification of significant target gene loci with higher resolution [21,22]. GWASs have been widely employed to dissect traits of interest for over a decade in soybean genetic research, including agronomic traits such as plant height, seed weight, flower, and pubescence color [23,24,25]; seed composition traits such as protein, oil, fatty acid, and amino acid contents [14,15,26]; and resistance to biotic stresses such as soybean aphids (*Aphis glycines* Matsumura) [27], bacterial blight (*Pseudomonas savastanoi* pv. *glycinea*) [28], bacterial leaf pustules (*Xanthornonas citri* pv. *glycines*) [29], frogeye leaf spot (*Cercospora sojina*) [30], brown spot [31], and soybean mosaic virus [13]. The present study aimed to (i) evaluate a large collection of *G. max* accessions for resistance to *P. sojae*, (ii) identify genomic locations and variations associated with resistance to *P*. *sojae* using multiple GWAS models, and (iii) discover candidate genes harbored in the causative genomic locations for *P. sojae* resistance.

## 2. Materials and Methods

### 2.1. Plant Materials and Experimental Design for a Large-Scale Phenotypic Assay

Seeds for 983 accessions (Table S1) of *G. max* germplasms were obtained from the National Agrobiodiversity Center, Wanju-gun, Jeollabuk-do, Republic of Korea, and evaluated for responses to *P. sojae* isolate 40468. Two Korean varieties, ‘Daepung’ [32] and ‘CheonAl’, [33] were used for susceptibility and resistance checks of *P. sojae* isolate 40468, respectively. For large-scale phenotypic assays, the 983 accessions were divided into 40 subsets, including ~25 accessions, the susceptible check ‘Daepung’, and the resistant check ‘CheonAl’. The two checks were included to verify inoculation success in all subsets of experiments. The experiment was repeated thrice. 

### 2.2. Evaluation of the Germplasm Collection for Resistance to P. sojae 

*P*. *sojae* isolate 40468 was isolated from Chungnam province in 1996 [34]. The isolate was originally obtained from the Korean Agricultural Culture Collection (KACC), National Agrobiodiversity Center, Rural Development Administration, Wanju-gun, Jeollabuk-do, South Korea. A single spore was re-isolated from the obtained sample, re-grown on V8 juice agar media, and stored in sterile water at 15 °C in the dark. From this in-house stock, the isolate was grown and used for each set of experiments. 

The reaction of each genotype to *P. sojae* was tested using a hypocotyl inoculation technique [35]. Briefly, 10–15 seeds of each accession were planted in a 13 cm plastic pot. *P. sojae* was grown on V8 juice agar media at 25 °C for 7 days. The V8 media fully covered with *P*. *sojae* mycelia were macerated using 50 mL syringes, and the mycelial slurry was transferred into a 10 mL syringe. A 1 cm incision was made on the hypocotyl under the cotyledon of 7-day-old seedlings using a scalpel. Then, 0.2–0.4 mL of mycelial slurry was injected into the incision. The inoculated seedlings were then placed under saturated humidity by covering them with plastic for 24 h and then translocated to a plant growth chamber for an additional 6 days. The environmental conditions inside the growth chamber were as follows: a 14:10 h day: night cycle, 25 °C, and >80% relative humidity. Seven days after inoculation, the reactions of each accession were determined based on the percentage of surviving plants from the inoculated total. Accessions were scored as resistant if 20% or fewer seedlings died and as susceptible if 80% or more seedlings died. Intermediate reactions were determined if the number of seedling deaths were between 20 and 80%. The phenotypic assays were repeated thrice.

### 2.3. Genotyping, Imputation, and Filtering of SNP Data

The accessions were genotyped using 180K Axiom SoyaSNP arrays (Affymetrix, Santa Clara, CA, U.S.) containing 169,028 recommended-quality SNPs distributed across the 20 soybean chromosomes. With 108,220 SNPs primarily filtered, imputation was performed to compensate for the missing genotypes using TASSEL 5.0 software (v5.5.86) [36], with default settings, except for the preferred haplotype size, max error to impute one donor, min site to test match, and min num of minor alleles to compare, which were set as 219, 872, 0.1, and 10, respectively. Further filtering after imputation eliminated 267 and 3318 SNPs due to >20% missing and a low minor allele frequency (MAF) of <0.01, respectively. A total of 104,635 SNPs were finally retained in the dataset and used for subsequent analyses (Table S2). 

### 2.4. Linkage Disequilibrium (LD) Decay Analysis 

LD decay was calculated using the software PopLDdecay v3.42 (https://github.com/BGI-shenzhen/PopLDdecay), with the following parameter set-up: MaxDist 1000, -MAF 0.01, and -Miss 0.2 [37]. The average *r*^2^ values were calculated for pairwise markers in a 1000 kb window, and these were then averaged across the euchromatic and heterochromatic regions, separately. The physical lengths of the euchromatic and heterochromatic regions in each chromosome were obtained from a previous study [38] (Table S3). Genome-wide LD was plotted as the physical distance (kb) versus *r*^2^ using PopLDdecay software v3.42 [37].

### 2.5. Genome-Wide Association Analysis and Haplotype Analysis

Genome-wide association analyses were performed with three different models using GAPIT3 software(https://github.com/jiabowang/GAPIT) [39], including the compressed mixed linear model (CMLM) [40], Bayesian-information and linkage-disequilibrium iteratively nested keyway (BLINK) [41], and fixed and random model circulating probability unification (FarmCPU) [42]. The thresholds of statistical significance (i.e., −log_10_*P* > 6.32) were determined by Bonferroni’s correction as α/no. of SNPs (α = 0.05, no. of SNP = 104,635). Manhattan and quantile–quantile plots were drawn to visualize each association. Gene annotation based on the reference Glyma.Wm82.a2.v1 was used to identify candidate genes within significantly associated loci. Annotated genes located in the LD block encompassing the significant SNPs identified by the GWAS were considered potential candidate genes for the target trait. 

### 2.6. Determination of LD Blocks for Genomic Regions Associated with P. sojae Resistance

Correlation coefficients (*r*^2^) of chromosome-wide SNP alleles were calculated to determine LD blocks using Haploview4.2 [43] with the following criteria: maximum distance, 500 kb; minimum minor allele frequency, 0.05; and Hardy–Weinberg cutoff, *p* < 0.05. LD blocks were determined if all four possible gametes between a pair of SNPs were observed with at least 5% frequency using the four-gamete method [44]. Adjacent blocks were combined if each block was separated by <10 kb [15,45]. 

The haplotypes of each LD block with 18 significant SNP markers were determined, and the haplotype frequencies of each LD block were calculated using Haploview4.2 [43]; only haplotypes with > 5% frequencies were considered in this analysis. These haplotype data were coupled with the distribution of phenotypic reactions among accessions. The number of accessions for each phenotypic reaction was counted by haplotype and visualized using R version 4.2.3 [46]. 

## 3. Results

### 3.1. Population Structure 

**Figure 1:**
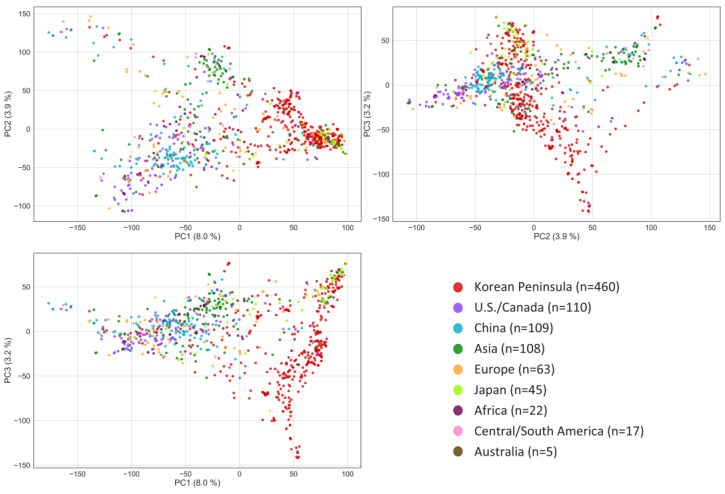
Genetic diversity of a panel of 983 soybean (*Glycine max* L. Merr.) accessions based on principal component analysis.

The GWAS panel comprised 983 accessions collected from at least 75 countries (Table S1). Most accessions originated from the Korean Peninsula (n = 460, 46.7%), followed by the U.S./Canada (n = 110, 11.2%), China (n = 109, 11.1%), and Japan (n = 45, 4.6%) (Table S1). Except for these countries of origin, some countries with relatively few germplasms were combined by continent or geographical region, such as Europe, Asia (Southeast), Africa, America (Central/South), and Australia, in the principal component analysis (PCA) scatter plot. The PCA showed that PC1, PC2, and PC3 explained 8.0, 3.9, and 3.2% of the observed variance, respectively, totaling approximately 15.1% explained by the first three PCs (Figure 1).

### 3.2. Phenotypic Distribution of Reactions Following Inoculation of P. sojae

**Figure 2:**
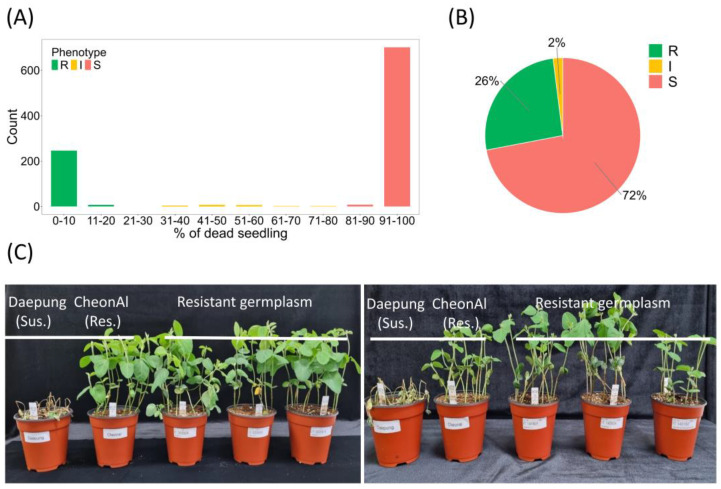
Phenotypic reactions to *Phytophthora sojae* isolate 40468 in the GWAS panel (n = 983): (**A**) Frequency distribution of the percentages of dead seedlings. (**B**) Segregation ratio of resistant (R), susceptible (S), and intermediate (I) reactions. (**C**) Phenotypic reactions of the Daepung (S check), CheonAl (R check), and selected resistant genotypes.

A collection of 983 soybean accessions were evaluated for resistance to *P*. *sojae* isolate 40468 using the hypocotyl inoculation technique. Two Korean varieties, ‘CheonAl’ and ‘Daepung’, were included as resistance and susceptibility checks in all experiment subsets to monitor the experimental conditions’ stability. CheonAl consistently exhibited resistance, whereas Daepung exhibited susceptibility. For the majority of individual accessions, phenotypic values were also highly consistent among replicated experiments based on the frequency distribution of standard deviations of the percentages of dead seedling (Figure S1). The percentage of dead seedlings ranged from 0 to 100% in the germplasm collection and showed a bimodal phenotypic distribution, with higher proportions at the two distal ends (Figure 2A). Of the 983 accessions, 253 (26%), 21 (2%), and 709 (72%) were resistant, intermediate, and susceptible, respectively (Figure 2B,C). 

### 3.3. Quality Control of 180K SNP Chip Data and Analysis of Linkage Disequilibrium

Of the initial 108,220 SNPs, 3585 SNPs were excluded by quality control (missing genotype rate >20% and minor allele frequency < 0.01), and 104,635 SNPs were used for subsequent analyses. These SNPs were evenly distributed across 20 chromosomes (Table S2; Figure S2). The average physical distance between the SNPs was 9.1 Kb, and the density of available SNPs ranged from 80 (chr. 1) to 135 (chr. 13) SNPs/megabase (Mb), with an average of 110 SNPs/Mb across the entire genome (Table S2). Figure S2 displays the genome-wide distribution and density of SNP within every 1Mb window.

The squared correlation coefficient (*r*^2^) was calculated for marker pairs. As the physical distance between two SNP locations increased, the *r^2^* values rapidly decreased. On average, across the entire genome, LD decayed to an *r*^2^ of 0.2 at approximately 85 kb and 1280 kb in the euchromatic and heterochromatic regions, respectively (Figure S3). 

### 3.4. Genome-Wide Association Analysis

**Figure 3:**
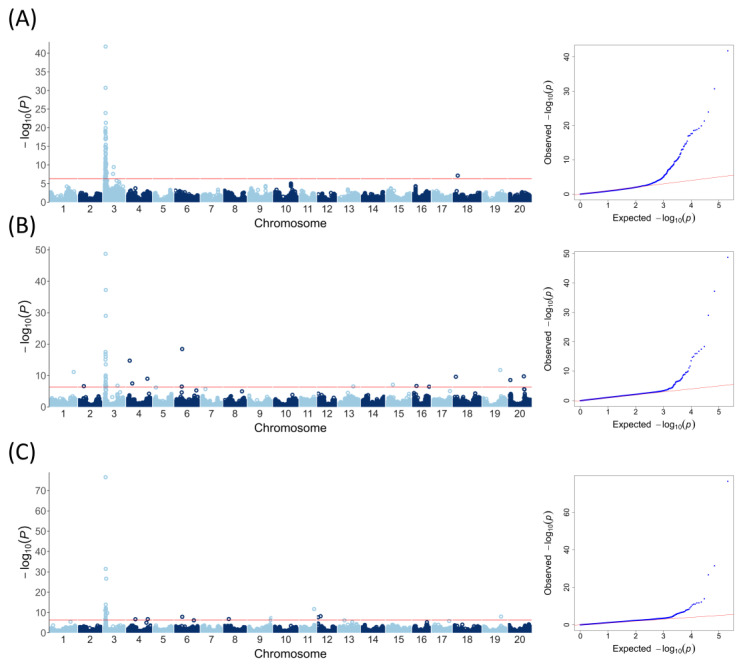
Manhattan plots (left) and QQ plots (right) for the genome-wide association study of the 983 soybean accessions for *P*. *sojae* resistance: (**A**) The compressed mixed linear (CMLM) model. (**B**) The Bayesian-information and linkage disequilibrium iteratively nested keyway (BLINK) model. (**C**) The fixed and random model circulating probability unification (FarmCPU) model.

High-throughput SNP genotypes and large-scale phenotypic assay results were coupled using advanced statistical methods to assess the genetic architecture of resistance to *P. sojae* isolate 40468, which originated in the Republic of Korea. Genome-wide association analyses were conducted using the phenotypic and genotypic data based on the three models, CMLM, Blink, and FarmCPU. In the quantile–quantile (QQ) plots of the CMLM, Blink, and FarmCPU, the observed −log_10_*P* distribution perfectly lay on a straight line until the expected −log_10_*P* was approximately three, but sharp deviations from the expected were observed at the tail of the straight line (i.e., −log_10_*P* > 3), indicating that these models successfully identified significant associations between SNPs and the phenotype, as shown in the Manhattan plots (Figure 3). The respective CMLM, BLINK, and FarmCPU models detected several to tens of SNPs significantly associated with the phenotypic results at the 5% genome-wide significance level (−log_10_ *P* = 6.32) (Table 1, Table 2 and Table 3). Using the CMLM, 77 of the 78 significant SNPs identified were located on chr. 3 with the highest levels of statistical significance, and only one SNP was identified on chr. 18 (Table 1). Unlike the CMLM, the BLINK and FarmCPU models identified additional loci on various chromosomes as well as many of the same SNPs identified on chr. 3 by the CMLM. Using the BLINK model, 22 SNPs were located on the same region of chr. 3, and the remaining 15 SNPs were found on nine other chromosomes (Table 2). Using the FarmCPU model, 18 of the 27 significant SNPs were detected on chr. 3, whereas the others detected on seven other chromosomes showed relatively lower significance (Table 3). Owing to the large number of significant SNPs identified, we attempted to select more reliable SNPs as those selected by at least two different models, for further analysis. As the list of significant SNPs identified by the BLINK and FarmCPU models did not completely overlap, the number of SNPs commonly identified by at least two models was 18, all of which were located on chr. 3. These SNPs were thus considered more reliable and significantly associated with resistance to *P*. *sojae* (Table S4, Figure S4). 

### 3.5. Haplotype Analysis for the Identified Genomic Region of Chromosome 3

**Figure 4:**
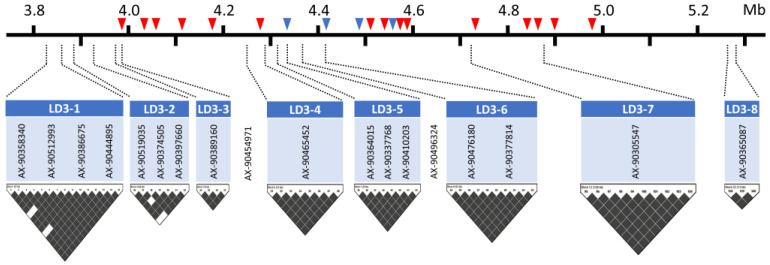
Linkage disequilibrium blocks, 18 consistently significant SNPs, and positions of annotated LRR and STK-coding genes within the genomic region of 3.8–5.3 Mb.

From genome-wide association analyses using the three models, a number of SNPs located in a wide range of chromosome 3 were identified; thus, the following haplotype analysis focused on the 3.8−5.3 Mb regions where the aforementioned 18 SNPs are positioned. Haplotype analysis determined eight LD blocks ranging from 8−156 kb in size in this interval; namely, LD3-1 to LD3-8 (Table 4, Figure 4). Sixteen SNPs were located in the eight LD blocks, while two significant SNPs (AX-90454971 at 3,988,688 bp, and AX-90496324 at 4,340,304 bp) were not included in any of the eight LD blocks. In each LD block, two to seven haplotypes were identified and named A to G (e.g., LD3-1-A); the highest haplotype frequency of each LD ranged from 21 to 56% (Table 4). Phenotypic distributions were investigated by haplotypes per LD block. In the majority of haplotypes across LD blocks, including the most frequent haplotypes in all eight LD blocks, the proportion of susceptible accessions was typically higher than that of resistant accessions. This finding is consistent with the observation that susceptible accessions accounted for three times the number of resistant genotypes in the entire panel (Table 4). Interestingly, certain haplotypes of LD3-2 (3,897,791−3,964,789 bp) and LD3-3 (3,982,340−3,990,383 bp) demonstrated markedly higher portions of resistance to *P. sojae*. It is noteworthy that 93–96% of the accessions with haplotypes LD3-2-F and -G exhibited resistance, as did haplotype LD3-3-C (Table 4). The frequencies of these haplotypes were 9%, 6%, and 9%, respectively, and were thus classified as rare haplotypes associated with resistance to *P. sojae*. Haplotype LD3-2-F was observed in 81 resistant accessions in LD3-3-C. Based on the reference genome (Glyma.Wm82.a2), LD3-3 partially overlaps with two predicted genes, of which one (Glyma.03G034200) is a nucleotide-binding site–leucine-rich repeat (NBS-LRR)-encoding gene known as a resistance gene in plants. 

## 4. Discussion

The identification of genetic sources for a target trait is a pivotal aspect of plant breeding, yet it remains a persistent challenge. Genome-wide association analysis is a powerful tool for detecting genomic regions associated with target traits using a large number of genotypes, obviating the necessity for the development of bi-parental segregating populations. In the present study, a total of 983 *G. max* accessions were screened for resistance to *P. sojae*, and many SNP markers on chr. 3 were identified with genome-wide significance for resistance to *P. sojae* isolate 40468 using the CMLM, BLINK, and FarmCPU models. The 18 SNPs in the 3.8–5.3 Mbp region on chr. 3 were consistently, highly significant by the three GWAS models, confirming the reliability of them (Table S4). In this region, the AX-90454971 (3,998,688 bp) exhibited the highest levels of significance (−log_10_*P* = 41.8−76.6) in the three models. The 3.8−5.3 Mbp interval is a well-known *Rps* cluster, with more than 20 *Rps* alleles previously identified from different resistance sources, including *Rps 1a, 1b, 1c, 7, UN1, YD29, YD25, HN, HC18,* unnamed alleles in cultivars Waseshiroge, Daewon, and Saedanbaek [9,47,48,49,50,51,52,53,54,55,56,57,58,59]. In soybean—*P*. *sojae* interactions, two major categories of disease resistance genes were highlighted as strong candidate genes in several previous studies: genes encoding NBS-LRR protein and serine/threonine kinase (STK) protein [53,54]. According to the reference genome (Glyma.Wm82.a2.v1), there are 88 annotated genes in the 3.8−5.3 Mbp interval, including 19 copies (>21%, 19/88) of NBS-LRR- and serine/threonine kinase-coding genes (Figure 4). A total of 15 LRR-coding genes are as follows: Glyma.03G034200, Glyma.03G034400, Glyma.03G034500, Glyma.03G064800, Glyma.03G034900, Glyma.03G035300, Glyma.03G037000, Glyma.03G037100, Glyma.03G037300, Glyma.03G037400, Glyma.03G038800, Glyma.03G039100, Glyma.03G039200, Glyma.03G039300, and Glyma.03G039500. In addition, Glyma.03G036000, Glyma.03G036500, Glyma.03G036900, and Glyma.03G037200 are the STK-coding genes. Such a high density of predicted *R* genes within this interval implies that this genomic region should be evolutionarily important and is often associated with broad-spectrum defense mechanisms in plants [60]. 

The clustering of *R* genes may be the result of unequal crossover, gene duplication, and gene conversion, which may have contributed to their functional significance in combating diverse pathogens [61]. Wide intraspecific variation can be found in copy numbers within clusters [62]. In *Arabidopsis*, 1439 crossovers within NBS-LRR clusters on chrs. 1 and 5 were reported and observed NBS-LRR genes associated with recombination hotspots, which were also detected as historical hotspots via an LD analysis of 260 *Arabidopsis* accessions [63]. The worldwide germplasm collection used in the current study is genetically diverse, and each accession should accumulate many historical recombination events in itself [20]. The average LD block size in the 3.8−5.3 Mbp region on chr. 3 was 43kb, which is smaller than the LD block size (approximately 85kb) averaged over euchromatic regions (Figure S3). The observed result suggests that this *R* gene cluster may have undergone a greater number of historical recombination than is typical. A high recombination rate within or between *R* genes has the potential to generate *R* gene structural and functional variations, resulting in a wide diversity of resistance sources among species [63]. In this *R* gene cluster, particular genes conferring resistance to the *P*. *sojae* isolate 40468 may vary among accessions; consequently, many markers anchored in a relatively wide genomic region (3.8–5.3 Mbp) on chr. 3 should be significantly associated. Hence, the introgression of multiple resistance genes from this important genomic region is a desirable long-term strategy in developing *P. sojae*-resistant varieties, as it could help decrease disease pressures by dominant *P. sojae* isolates in soybean fields.

In the present study, the CMLM identified 77 significant SNPs on chr. 3 and 1 SNP on chr. 18. Unlike the CMLM, the BLINK and FarmCPU models demonstrated meaningful performances by identifying 37 and 11 SNPs on eleven and eight different chromosomes, respectively. It is noteworthy that the FarmCPU and BLINK identified 24 additional SNPs on different chromosomes in addition to the 3.8–5.3 Mbp on chr. 3, including several new genomic regions as well as those similar to the already known regions. The BLINK model also identified additional significant SNPs on other chrs. 13, 15, 16, and 20, where resistance to *P. sojae* was previously reported. On chr. 13, one significant SNP (10.4 Mbp) identified as overlapping with the *Rps*3 region [50]. Quantitative disease-resistant loci (QDRL) for partial resistance to *P*. *sojae* have been documented in adjacent locations [64,65,66]. A SNP positioned at 51,347,154 bp on chr. 1 was found to be new and highly significant (−log_10_*P* = 11.1), which minor allele ‘T’ contributes to resistance. The FarmCPU model identified a significant locus on chr. 6, where a QDRL for *P*. *sojae* was identified [64]. AX-90510915 (at 5980,832 bp on chr. 12) is also novel and highly significant (−log_10_*P* = 8.2), of which the minor allele ‘T’ conferred resistance to *P*. *sojae*. These findings indicate that the BLINK and FarmCPU models can enhance the capabilities of the single-locus model by identifying genomic regions with minor effects. From these results, the combination of these rare alleles can facilitate the attainment of enhanced resistance.

The CMLM is employed for single-locus analysis, which enhances the computational capabilities and statistical power in comparison to the previously described MLM method [40]. MLM methods can hardly detect other low-effect significant markers for complex traits, as they focus on large-effect markers [42,67]. In contrast, the BLINK and FarmCPU models identified significant SNPs on different chromosomes that were not detected by the CMLM. They are capable of identifying additional markers with smaller effects that may be overlooked by the single-locus method. Furthermore, the CMLM identified many significant SNPs on chr. 3 that were in close proximity. In contrast, the BLINK and FarmCPU models reduced the number of identified SNPs to 22 or fewer. This enables the more precise detection of markers that can be employed in marker-assisted selection and genomic selection.

All the MLM methods are single-locus models that test one marker at a time. Consequently, they are prone to an increased number of false negatives. To address this issue, several multi-locus models have been developed and utilized in GWASs, including FASTmrEMMAa and FASTmrMLM [68], ISIS EM-BLASSO [69], pLARmEB [70], pKWmEB [71], LASSO [72], and others [73]. FarmCPU and BLINK are multi-locus models that are capable identifying both major- and minor-effect markers, thereby increasing the proportion of genetic variance [67]. The FarmCPU model employes an iterative process that utilizes both fixed and random models with the most significant markers as covariates. This approach helps to prevent overfitting, reduces the number of reported significant markers, and effectively controls for false positives and negatives. The BLINK model is superior in statistical power with the discovery of fewer false positives than other models because it eliminates the assumption that causal genes are uniformly distributed across the genome [41]. In a GWAS for stem termination habits in soybean [74], the CMLM identified multiple significant SNP markers at close physical distances on the same chromosome, while FarmCPU identified additional significant SNPs on other chromosomes [74]. Similar findings were observed in a GWAS for seedling emergence in wheat [67], where MLM identified numerous neighboring SNPs, predominantly on chr. 5. However, the FarmCPU and BLINK methods identified multiple SNPs on different chrs. 1, 2, 5, 6, and 7, with relatively modest effects and *R^2^* values. This highlights the capacity of multi-locus models to detect small-effect markers.

Interestingly, the present study identified a diverse range of *Rps* loci that conferred resistance to the same *P. sojae* isolate 40468, rather than the previously documented genomic region (55.9−56.4 Mbp) on chr. 18. Phenotypic reactions are the result of the interaction between *Rps* genes and cognate *Avr* genes, which can occur in multiple ways. The current dataset does not allow us to directly address the question of why the *Rps* cluster located on chr. 18 was not identified in the present study. A potential explanation is that the two *R* loci exhibited functional redundancy or overlapping recognition of the same *Avr* genes, and the *Rps* genes underlying chr. 3 exhibited a response to cognate *Avr* genes in the isolate 40468 in the majority of the accessions, resulting in a statistically significant outcome in GWAS. Conversely, this may obscure the statistical significance of *Rps* on chr. 18 in a subset of the accessions. It is possible for multiple *R* genes to be involved in resistance to the same *Avr* gene. For example, in the case of *Brassica napus*, the *AvrLm4-7* gene of *Leptosphaeria maculans* is recognized by two *R* genes, *Rlm4* and *Rlm7*, both of which contribute to resistance against the pathogen [75]. However, the extensive use of cultivars containing *Rlm7* led to the emergence of new strains of the pathogen that were able to evade the resistance provided by the *Rlm7*, thereby demonstrating the evolutionary pressure that pathogens exert on single *R* gene-mediated resistance [76,77,78]. In rare instances, point mutations in the pathogen’s *Avr* genes can result in virulence against multiple *R* genes due to dual specificity, even in the absence of widespread *R* gene deployment [77]. It is therefore evident that the incorporation of redundant *R* genes into breeding programs is of significant value, as they serve as a safeguard against resistance breakdown caused by the pathogen’s adaptation [79].

It is thus recommended that the various *Rps* genes be identified on an ongoing basis and introduced into the relevant cultivars to achieve more durable or resilient resistance to *P. sojae*. The alteration in pathotypes of U.S. *P*. *sojae* populations demonstrated that the proportion of pathotypes capable of overcoming specific *Rps* genes increased over a few decades. Consequently, some *Rps* genes are no longer capable of providing effective protection [6,75]. In view of the adaptation of *P. sojae* to *Rps* genes and the limited longevity of *Rps* genes [12], the redundant genetic sources are of importance. The use of alternative or diverse *Rps* genes can effectively reduce selection pressures for particular *Avr* genes. With this consideration, the pyramiding of multiple *R* genes can provide the robustness and durability of resistance by creating more complex barriers against the pathogen, thereby overcoming it. [33]. The findings of this study indicate that at least two distinct *R* gene loci may provide effective protection against *P. sojae* isolate 40468, potentially through functional redundancy or complementary resistance mechanisms.

In conclusion, the phenotypic data of 983 *G. max* accessions were derived from hypocotyl inoculation assays using *P. sojae* isolate 40468. Genotypic data from a diverse panel of soybean accessions, representing a broad spectrum of genetic diversity, were integrated with phenotypic results to identify resistance loci via a GWAS. A considerable number of SNPs are located within the known *Rps* region on chr. 3, as they are significantly associated with the resistance. Haplotype analyses demonstrated that a few haplotypes (LD3-2-F and LD3-3-C) exhibited enhanced concordance between the haplotype and phenotypic reactions. Moreover, a few new nucleotide variations associated with the increased resistance on chr. 1 and 12 will contribute to the current understanding of *Rps*, which requires further investigation. The identified genomic variations and predicted candidate genes have the potential to enhance our understanding of the molecular mechanisms governing soybean-*P. sojae* interactions, thereby facilitating the development of more effective and sustainable disease management strategies during soybean cultivation. Overall, this study provides a foundation for future research aimed at enhancing soybean resilience to *P. sojae* and other related pathogens.

## Figures and Tables

**Table 1:** Significant SNPs identified by the CMLM for resistance to *Phytophthora sojae* isolate 40468.

Chr ^a^	SNP ID	Marker Position(bp) ^b^	Allele ^c^	MAF ^d^	−log10(*P*) ^e^	R² (%) ^f^	Allelic Effect ^g^
3	AX-90452294	1,983,038	G/T	0.25	6.5	1.3	8.8
3	AX-90471572	3,010,747	T/C	0.05	7.1	1.4	18.0
3	AX-90375448	3,015,875	A/G	0.04	6.9	1.4	19.4
3	AX-90478226	3,053,066	G/A	0.03	8.8	1.8	22.7
3	AX-90365805	3,110,523	T/C	0.04	6.6	1.3	18.8
3	AX-90448600	3,403,744	C/T	0.33	7.4	1.5	9.6
3	AX-90372396	3,403,812	A/G	0.33	7.4	1.5	9.6
3	AX-90380038	3,423,161	C/T	0.45	8.7	1.8	−9.9
3	AX-90345784	3,429,332	G/A	0.10	14.9	3.2	17.5
3	AX-90318187	3,448,160	A/G	0.46	7.1	1.4	−7.6
3	AX-90524133	3,458,537	T/C	0.45	10.3	2.1	−10.8
3	AX-90378648	3,484,755	G/T	0.45	9.8	2.0	−9.7
3	AX-90420887	3,503,677	C/A	0.18	19.2	4.2	16.7
3	AX-90515540	3,504,993	C/T	0.28	17.0	3.7	14.3
3	AX-90449575	3,517,886	A/C	0.47	9.5	1.9	10.3
3	AX-90517913	3,519,952	A/G	0.15	11.7	2.4	−15.0
3	AX-90438931	3,542,850	G/C	0.19	10.9	2.3	−16.1
3	AX-90388258	3,554,469	T/A	0.34	7.9	1.6	10.0
3	AX-90497688	3,554,849	G/A	0.11	13.9	2.9	16.3
3	AX-90377747	3,637,876	C/T	0.14	9.6	2.0	12.2
3	AX-90334631	3,638,108	G/A	0.14	9.6	2.0	12.2
3	AX-90319861	3,652,555	C/T	0.38	7.1	1.4	-8.9
3	AX-90442897	3,652,881	C/T	0.38	7.1	1.4	−8.9
3	AX-90475860	3,653,078	A/C	0.19	18.8	4.1	16.1
3	AX-90378751	3,655,668	G/A	0.30	7.8	1.6	−11.8
3	AX-90440228	3,759,276	C/T	0.16	10.7	2.2	15.7
3	AX-90329739	3,788,845	T/A	0.19	8.6	1.8	10.9
3	AX-90368467	3,820,234	G/A	0.41	8.1	1.6	−9.9
3	AX-90477435	3,820,849	G/A	0.12	6.6	1.3	−14.4
3	AX-90358340	3,826,007	G/A	0.45	18.6	4.1	14.6
3	AX-90512993	3,826,375	T/C	0.45	18.6	4.1	14.5
3	AX-90386675	3,836,189	T/C	0.14	12.0	2.5	18.0
3	AX-90444895	3,855,796	T/C	0.09	8.4	1.7	−18.0
3	AX-90488574	3,859,704	T/C	0.09	8.1	1.6	−17.6
3	AX-90519035	3,897,791	G/T	0.22	24.0	5.3	21.5
3	AX-90375813	3,904,817	G/A	0.22	7.4	1.5	−11.7
3	AX-90374505	3,911,731	C/T	0.11	16.9	3.7	22.6
3	AX-90320182	3,953,294	G/A	0.11	30.7	7.0	29.2
3	AX-90377480	3,962,150	T/C	0.02	13.0	2.7	36.0
3	AX-90397660	3,964,789	C/T	0.22	19.9	4.4	19.6
3	AX-90370699	3,973,776	A/G	0.33	9.9	2.0	11.3
3	AX-90356748	3,982,340	C/T	0.21	11.5	2.4	−13.9
3	AX-90317982	3,988,291	G/C	0.07	10.9	2.3	23.0
3	AX-90389160	3,989,908	T/A	0.32	13.1	2.8	13.6
3	AX-90454971	3,998,688	G/A	0.18	41.8	9.9	25.5
3	AX-90456806	4,111,692	C/A	0.33	8.1	1.6	−9.9
3	AX-90417885	4,272,521	G/A	0.31	6.5	1.3	−7.8
3	AX-90465452	4,283,885	T/A	0.21	17.7	3.8	−15.9
3	AX-90331552	4,291,232	T/C	0.28	9.4	1.9	11.3
3	AX-90307867	4,291,566	A/T	0.28	9.6	2.0	11.5
3	AX-90312261	4,312,581	T/A	0.22	10.1	2.1	−12.3
3	AX-90364015	4,318,337	G/A	0.10	8.4	1.7	15.9
3	AX-90322967	4,325,128	A/G	0.18	14.6	3.1	−15.4
3	AX-90337768	4,325,664	G/A	0.30	7.1	1.4	−8.4
3	AX-90385376	4,328,363	G/A	0.30	7.3	1.5	−8.6
3	AX-90410203	4,338,933	A/G	0.04	14.1	3.0	28.3
3	AX-90496324	4,340,304	A/G	0.16	15.5	3.3	18.1
3	AX-90476180	4,340,465	A/C	0.10	12.8	2.7	19.5
3	AX-90402524	4,355,566	C/T	0.34	7.7	1.5	−10.4
3	AX-90377814	4,356,151	T/G	0.03	8.0	1.6	28.6
3	AX-90504759	4,405,356	A/T	0.43	21.3	4.7	14.5
3	AX-90306896	4,414,031	T/C	0.43	17.6	3.8	−11.9
3	AX-90456732	4,466,635	A/C	0.34	13.1	2.8	11.4
3	AX-90314290	4,639,308	A/T	0.13	9.7	2.0	13.5
3	AX-90393523	4,642,893	T/C	0.45	7.4	1.5	−8.5
3	AX-90509372	4,717,222	C/T	0.39	6.9	1.4	8.4
3	AX-90511516	4,752,915	T/C	0.32	7.4	1.5	8.3
3	AX-90305547	4,860,134	G/A	0.04	17.1	3.7	36.6
3	AX-90392159	5,024,763	T/C	0.47	7.0	1.4	8.1
3	AX-90317989	5,120,075	A/G	0.34	14.6	3.1	14.3
3	AX-90495242	5,205,514	C/A	0.48	8.3	1.7	9.1
3	AX-90365087	5,287,030	G/T	0.23	10.4	2.2	11.3
3	AX-90351533	5,307,926	G/C	0.09	8.3	1.7	17.6
3	AX-90306686	5,353,786	T/C	0.11	10.1	2.1	−13.6
3	AX-90449871	6,697,315	G/T	0.05	7.8	1.6	18.9
3	AX-90353442	20,609,695	A/G	0.01	7.6	1.5	26.9
3	AX-90480626	21,957,219	G/A	0.06	9.4	1.9	−22.2
18	AX-90414741	8,261,882	T/A	0.38	7.2	1.4	7.7

^a^ Chromosome; ^b^ physical positions (bp) are based on the latest soybean reference genome (Glyma.Wm82.a2); ^c^ major/minor allele; ^d^ minor allele frequency; ^e^ statistical significance (*p*-value) after modified Bonferroni’s correction of the identified SNP; ^f^ (R^2^ of the model with the SNP − R^2^ of the model without the SNP) × 100; ^g^ and the effect of the major allele relative to the minor allele by SNP.

**Table 2:** Significant SNPs identified by the BLINK model for resistance to *Phytophthora sojae* isolate 40468.

Chr ^a^	SNP ID	Marker Position (bp) ^b^	Allele ^c^	MAF ^d^	−log10(*P*) ^e^	R^2^ (%) ^f^	Allelic Effect ^g^
1	AX-90452255	51,347,154	G/T	0.01	11.1	3.0	−19.7
2	AX-90351632	10,409,311	C/A	0.42	6.6	0.1	2.6
3	AX-90324356	3,159,070	G/A	0.12	6.8	0.2	5.2
3	AX-90472606	3,638,005	T/G	0.46	7.2	0.3	4.9
3	AX-90482211	3,741,175	G/A	0.01	15.1	19.5	−27.9
3	AX-90444895	3,855,796	T/C	0.09	8.0	0.0	−15.8
3	AX-90498561	3,893,390	A/G	0.27	6.6	0.1	5.7
3	AX-90519035	3,897,791	G/T	0.22	17.5	1.3	10.8
3	AX-90374505	3,911,731	C/T	0.11	16.8	1.8	10.7
3	AX-90397660	3,964,789	C/T	0.22	16.0	1.0	9.6
3	AX-90389160	3,989,908	T/A	0.32	9.6	0.5	7.3
3	AX-90454971	3,998,688	G/A	0.18	48.7	5.0	18.5
3	AX-90503215	4,200,520	A/G	0.41	10.0	0.7	−6.5
3	AX-90310078	4,277,380	T/C	0.40	7.6	0.9	−7.3
3	AX-90465452	4,283,885	T/A	0.21	16.0	0.9	−11.7
3	AX-90317436	4,295,128	A/G	0.49	29.0	3.1	20.0
3	AX-90337768	4,325,664	G/A	0.30	6.5	0.3	−5.3
3	AX-90410203	4,338,933	A/G	0.04	13.5	4.0	14.7
3	AX-90496324	4,340,304	A/G	0.16	8.8	0.5	6.6
3	AX-90377814	4,356,151	T/G	0.03	37.2	4.3	29.1
3	AX-90305547	4,860,134	G/A	0.04	8.9	3.0	14.0
3	AX-90502539	5,261,082	G/A	0.21	6.5	0.2	−4.5
3	AX-90365087	5,287,030	G/T	0.23	6.7	0.2	4.3
3	AX-90436811	30,366,251	A/T	0.07	6.8	0.8	−5.3
4	AX-90469303	5,395,490	T/C	0.09	14.7	0.4	9.0
4	AX-90453638	11,057,625	G/A	0.19	7.5	0.2	4.1
4	AX-90314529	44,548,176	T/C	0.42	9.0	0.2	−3.3
6	AX-90386189	13,985,551	G/A	0.01	6.5	2.1	−12.5
6	AX-90396263	14,955,621	C/A	0.40	18.4	0.3	−5.6
13	AX-90344339	32,713,320	T/A	0.27	6.5	0.1	−2.8
15	AX-90512796	13,188,114	T/C	0.02	7.1	2.6	−12.1
16	AX-90516316	8,045,311	T/C	0.02	6.7	1.0	10.7
16	AX-90409602	36,074,874	A/G	0.01	6.5	2.5	−10.9
18	AX-90307453	4,083,239	T/A	0.03	9.6	0.7	9.3
19	AX-90441950	37,671,280	C/T	0.05	11.8	0.9	−10.3
20	AX-90446295	2,937,531	T/C	0.01	8.6	0.7	11.1
20	AX-90509320	32,927,057	T/C	0.02	9.7	1.0	−14.5

^a^ Chromosome; ^b^ physical positions (bp) are based on the latest soybean reference genome (Glyma.Wm82.a2); ^c^ major/minor allele; ^d^ minor allele frequency; ^e^ statistical significance (*p*-value) after modified Bonferroni’s correction of the identified SNP; ^f^ (R^2^ of the model with the SNP − R^2^ of the model without the SNP) × 100; ^g^ and the effect of the major allele relative to the minor allele by SNP.

**Table 3:** Significant SNPs identified by the FarmCPU model for resistance to *Phytophthora sojae* isolate 40468.

Chr ^a^	SNP ID	Marker Position (bp) ^b^	Allele ^c^	MAF ^d^	−log10(*P*) ^e^	R^2^(%) ^f^	Allelic Effect ^g^
3	AX-90493588	3,208,379	A/C	0.03	11.0	3.0	16.2
3	AX-90358340	3,826,007	G/A	0.45	7.1	0.2	4.9
3	AX-90512993	3,826,375	T/C	0.45	7.0	0.0	4.9
3	AX-90386675	3,836,189	T/C	0.14	11.1	0.6	9.1
3	AX-90476839	3,847,486	G/A	0.03	6.5	1.1	12.0
3	AX-90374505	3,911,731	C/T	0.11	6.4	0.7	8.3
3	AX-90389160	3,989,908	T/A	0.32	6.5	0.5	5.3
3	AX-90454971	3,998,688	G/A	0.18	76.6	15.8	25.4
3	AX-90328472	4,284,091	C/T	0.12	31.5	8.6	−15.3
3	AX-90408663	4,291,736	G/A	0.10	6.6	3.7	7.5
3	AX-90364015	4,318,337	G/A	0.10	8.3	1.3	10.4
3	AX-90496324	4,340,304	A/G	0.16	9.1	1.1	7.2
3	AX-90476180	4,340,465	A/C	0.10	10.6	2.6	9.8
3	AX-90349616	4,348,187	C/T	0.08	13.9	3.0	12.5
3	AX-90377814	4,356,151	T/G	0.03	12.2	2.0	19.5
3	AX-90305547	4,860,134	G/A	0.04	11.7	2.6	20.7
3	AX-90365087	5,287,030	G/T	0.23	26.7	1.1	11.1
3	AX-90361277	7,600,310	G/A	0.11	9.8	0.7	−7.5
4	AX-90305713	17,966,319	T/C	0.16	6.7	4.2	−6.2
4	AX-90341073	45,436,256	G/A	0.46	6.8	0.1	−4.5
6	AX-90522264	15,060,984	G/A	0.21	7.9	0.6	5.6
8	AX-90371996	10,267,323	T/C	0.08	6.8	0.7	8.5
9	AX-90395828	49,981,866	C/T	0.05	7.2	0.9	9.4
11	AX-90425598	31,549,372	C/A	0.03	11.7	3.6	−15.4
12	AX-90471689	684,016	T/C	0.04	7.7	29.9	8.8
12	AX-90510915	5,980,832	C/T	0.02	8.2	1.9	−16.5
19	AX-90379903	39,419,993	G/A	0.06	8.0	0.3	9.7

^a^ Chromosome; ^b^ physical positions (bp) are based on the latest soybean reference genome (Glyma.Wm82.a2); ^c^ major/minor allele; ^d^ minor allele frequency; ^e^ statistical significance (*p*-value) after modified Bonferroni’s correction of the identified SNP; ^f^ (R^2^ of the model with the SNP − R^2^ of the model without the SNP) × 100; ^g^ and the effect of the major allele relative to the minor allele by SNP.

**Table 4:** Haplotypes and phenotypic segregation by haplotype in linkage disequilibrium blocks of 3.8–5.3 Mbp.

Linkage Disequilibrium (LD)			Haplotype ID	Haplotype ^a^															Frequency	Numbers of Accession by Reaction		
Block	Interval	Size (Kb)																		Resistant	Susceptible	Intermediate
LD3-1	3,826,007–3,893,390	67	Haplotype A	G	T	T	G	A	A	T	G	A	C	T	T	C	T	G	21%	7 (3%)	202 (96%)	2 (1%)
			Haplotype B	G	T	T	G	A	A	T	G	A	T	T	T	C	T	A	17%	31 (19%)	131 (80%)	2 (1%)
			Haplotype C	A	C	T	G	A	A	T	G	A	C	T	T	C	T	A	14%	78 (57%)	52 (38%)	8 (6%)
			Haplotype D	A	C	T	G	G	G	A	G	A	T	T	T	C	T	A	11%	19 (17%)	91 (82%)	1 (1%)
			Haplotype E	A	C	C	G	A	G	T	G	A	T	T	T	A	T	A	10%	42 (45%)	51 (54%)	1 (1%)
			Haplotype F	G	T	T	G	A	G	T	A	A	T	C	C	C	T	A	9%	81 (95%)	2 (2%)	2 (2%)
LD3-2	3,897,791–3,964,789	66	Haplotype A	G	G	C	T	G	T	C									32%	26 (8%)	289 (91%)	3 (1%)
			Haplotype B	G	G	C	C	G	T	C									11%	12 (11%)	96 (88%)	1 (1%)
			Haplotype C	T	G	C	T	G	T	C									11%	3 (3%)	99 (95%)	2 (2%)
			Haplotype D	G	A	C	C	G	T	C									7%	4 (6%)	68 (93%)	1 (1%)
			Haplotype E	G	A	C	T	G	T	T									9%	14 (16%)	69 (78%)	5 (6%)
			Haplotype F	T	G	C	T	A	T	T									9%	81 (96%)	0 (0%)	3 (4%)
			Haplotype G	G	G	T	C	G	T	C									6%	52 (93%)	3 (5%)	1 (2%)
LD3-3	3,982,340–3,990,383	8	Haplotype A	C	G	T	G												56%	76 (14%)	470 (85%)	8 (1%)
			Haplotype B	T	G	A	G												16%	34 (21%)	124 (78%)	1 (1%)
			Haplotype C	C	G	A	G												9%	82 (95%)	0 (%)	4 (5%)
			Haplotype D	C	G	T	A												7%	5 (8%)	53 (83%)	6 (9%)
			Haplotype E	C	C	A	G												7%	47 (73%)	17 (27%)	0 (0%)
LD3-4	4,271,895–4,284,091	12	Haplotype A	C	G	T	A	A	T	T	C								53%	113 (22%)	393 (76%)	14 (3%)
			Haplotype B	C	A	C	A	A	C	A	C								9%	3 (3%)	82 (94%)	2 (2%)
			Haplotype C	C	A	C	A	A	C	A	T								8%	14 (17%)	67 (82%)	1 (1%)
			Haplotype D	C	G	C	A	A	C	T	C								8%	31 (38%)	49 (60%)	2 (2%)
			Haplotype E	C	A	C	A	A	C	T	C								8%	53 (70%)	23 (30%)	0 (0%)
LD3-5	4,318,337–4,339,042	20	Haplotype A	G	G	A	G	G	T	A	A								60%	113 (19%)	463 (79%)	14 (2%)
			Haplotype B	G	G	G	A	A	T	A	A								13%	18 (14%)	111 (84%)	3 (2%)
LD3-6	4,340,465–4,403,158	62	Haplotype A	A	C	C	C	T	T	G	T	T	G						38%	30 (8%)	333 (89%)	11 (3%)
			Haplotype B	A	C	C	C	T	T	G	T	C	G						19%	87 (47%)	99 (53%)	1 (1%)
			Haplotype C	A	C	C	T	T	T	A	C	C	G						14%	16 (12%)	115 (87%)	2 (2%)
LD3-7	4,717,222–4,873,970	156	Haplotype A	C	T	A	C	A	T	A	C	G	G						42%	64 (15%)	345 (83%)	6 (1%)
			Haplotype B	T	C	G	C	A	T	A	C	G	G						15%	53 (35%)	91 (61%)	6 (4%)
			Haplotype C	T	C	G	C	A	T	A	C	G	T						7%	29 (41%)	40 (57%)	1 (1%)
			Haplotype D	C	T	A	C	A	T	G	T	G	G						9%	5 (6%)	78 (92%)	2 (2%)
LD3-8	5,273,348–5,287,030	13	Haplotype A	T	A	G													35%	70 (20%)	267 (77%)	9 (3%)
			Haplotype B	C	G	G													40%	73 (18%)	315 (79%)	9 (2%)
			Haplotype C	C	G	T													23%	108 (49%)	112 (51%)	2 (1%)

^a^ Different colors indicate different nucleotides.

## Data Availability

The original contributions presented in this study are included in this article/Supplementary Materials, and further inquiries can be directed to the corresponding author/s.

## Supplementary Materials

The following supporting information can be downloaded at https://www.mdpi.com/article/10.3390/plants13243501/s1, Figure S1. Frequency distribution of standard deviations of the percentages of dead seedlings among replications of individuals; Figure S2. Chromosome-wide distributions and the densities of SNP in every 1Mb window. The horizontal axis represents chromosome length (in mega base pair), and different bar colors indicate varying levels of SNP density; Figure S3. Linkage disequilibrium (LD) decay rates in euchromatic and heterochromatic regions. Linkage disequilibrium (*r*^2^) was estimated by using all pairs of single nucleotide polymorphisms within 1000 kb of physical distance in the euchromatic and heterochromatic regions of 983 soybean accessions; Figure S4. A Venn diagram for overlapping and independent gene numbers among the three GWAS models; Table S1. A collection of 983 soybean (*Glycine max* L.) genotypes used in the present study; Table S2. Numbers and the density of SNP per chromosome; Table S3. Pericentromeric regions of 20 chromosomes of soybean according to Schmutz et al. [38]; Table S4. SNPs significantly associated with resistance to *Phytophthora sojae* isolate 40468 consistently identified by the CMLM, BLINK, and FarmCPU models.
