# Supplementary figures and images for: Genome-Wide Association Study for Resistance to Phytophthora sojae in Soybean [Glycine max (L.) Merr.]
Source: Plants (Basel). 2024 Dec 15;13(24):3501. doi: 10.3390/plants13243501 (PMC11676158; doi:10.3390/plants13243501)

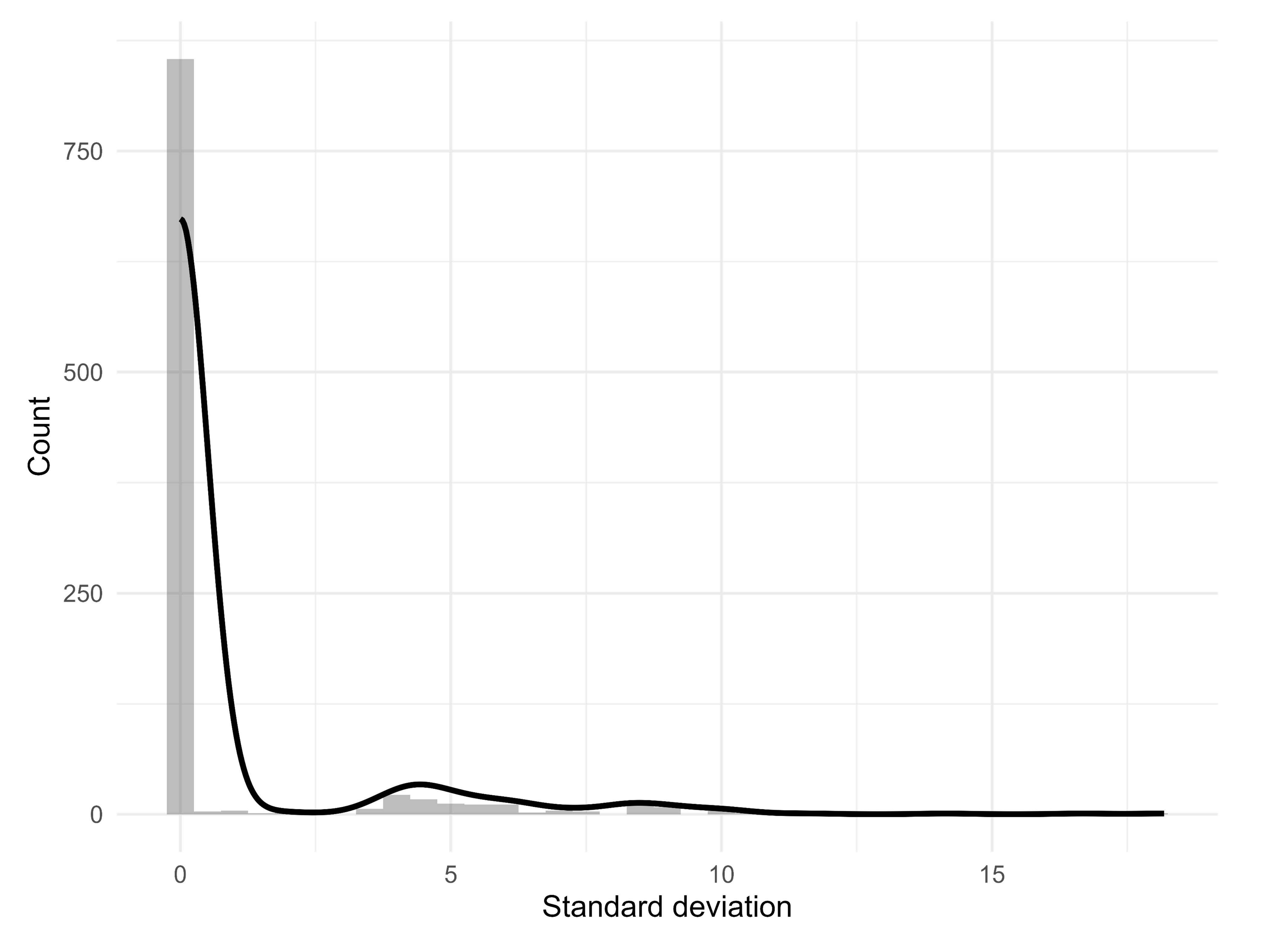

Supplement: Supplementary file 1 [file plants-13-03501-s001.zip › FIgure S1.tif]

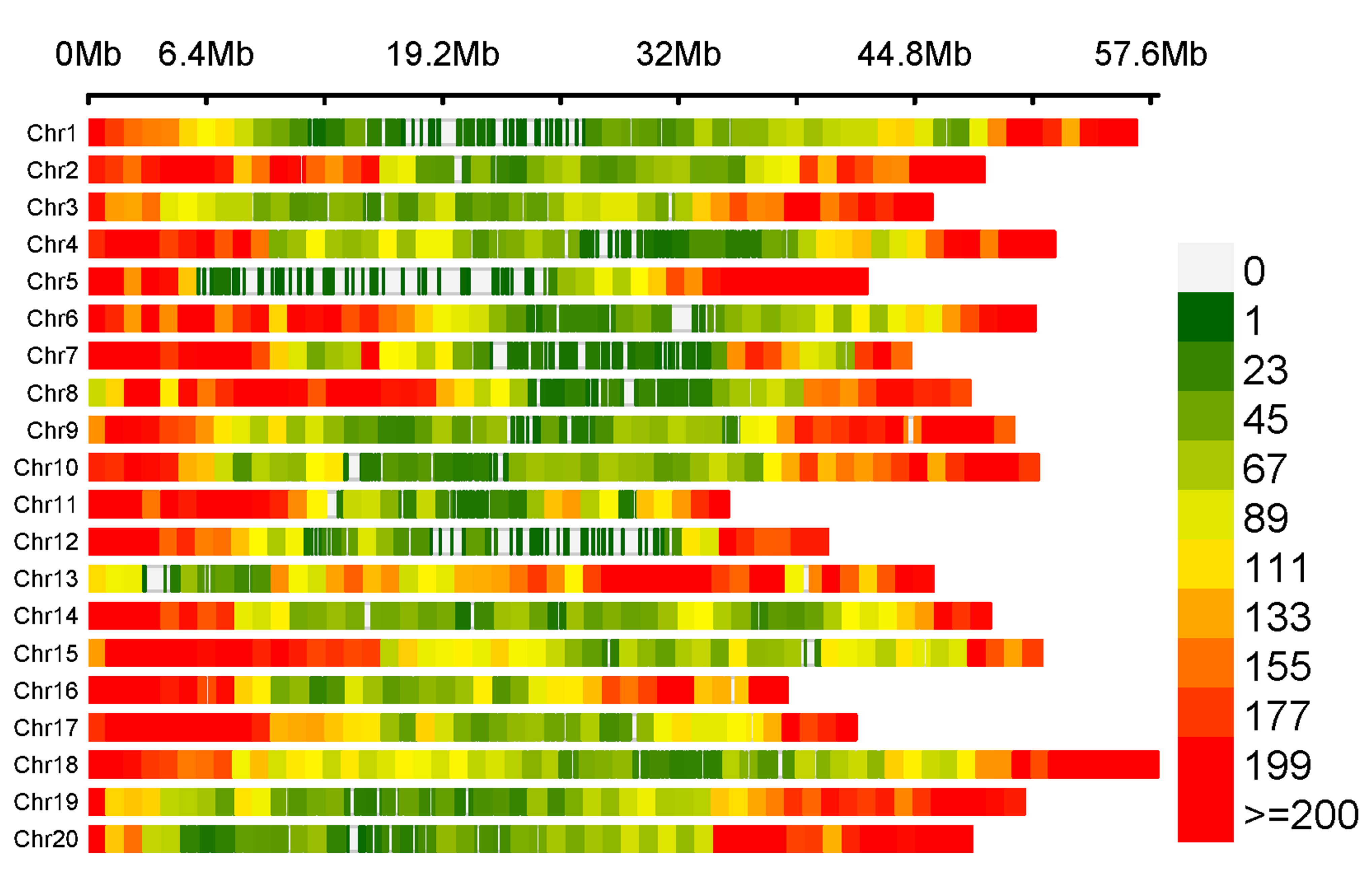

Supplement: Supplementary file 1 [file plants-13-03501-s001.zip › FIgure S2.tif]

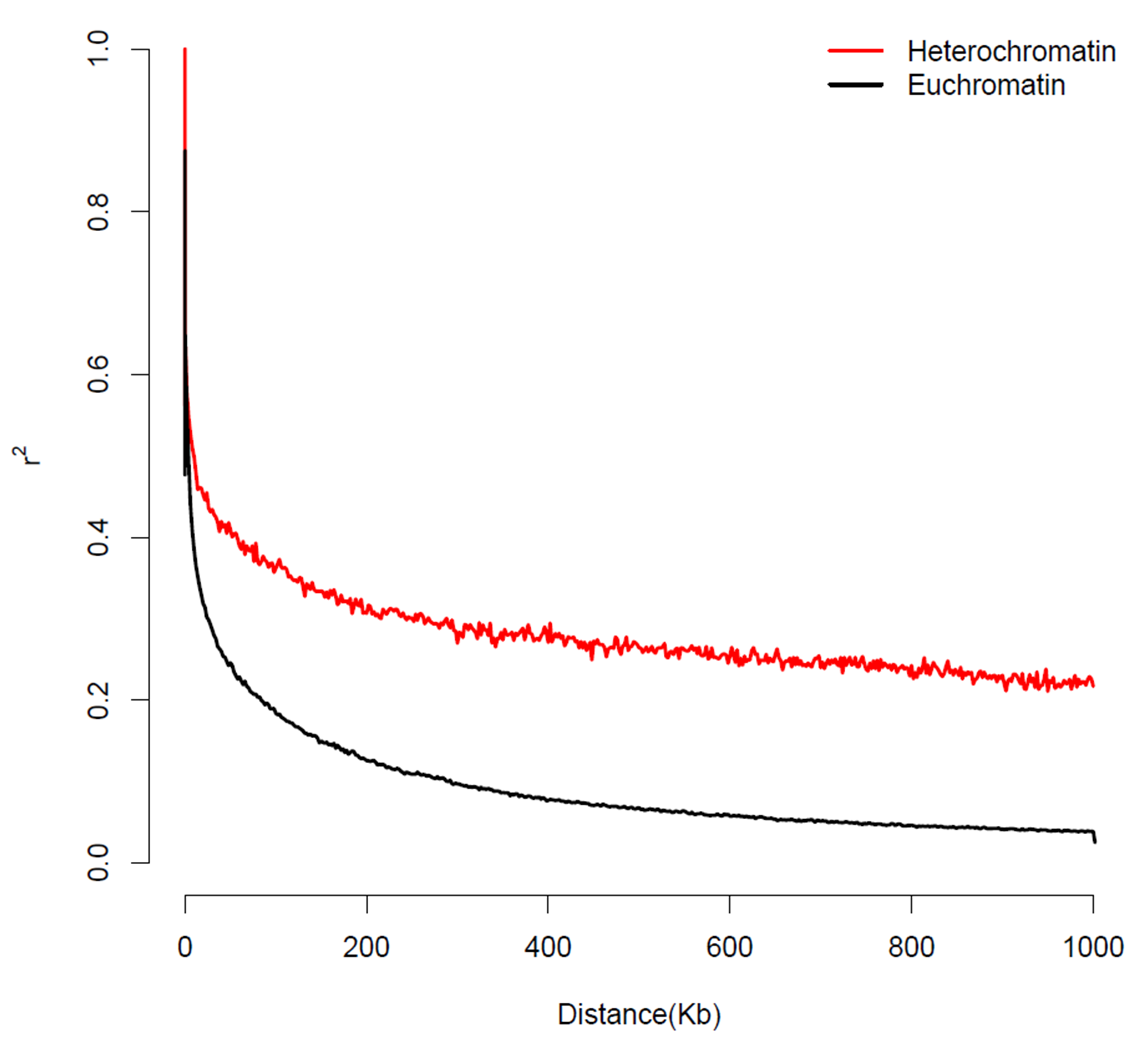

Supplement: Supplementary file 1 [file plants-13-03501-s001.zip › FIgure S3.TIF]

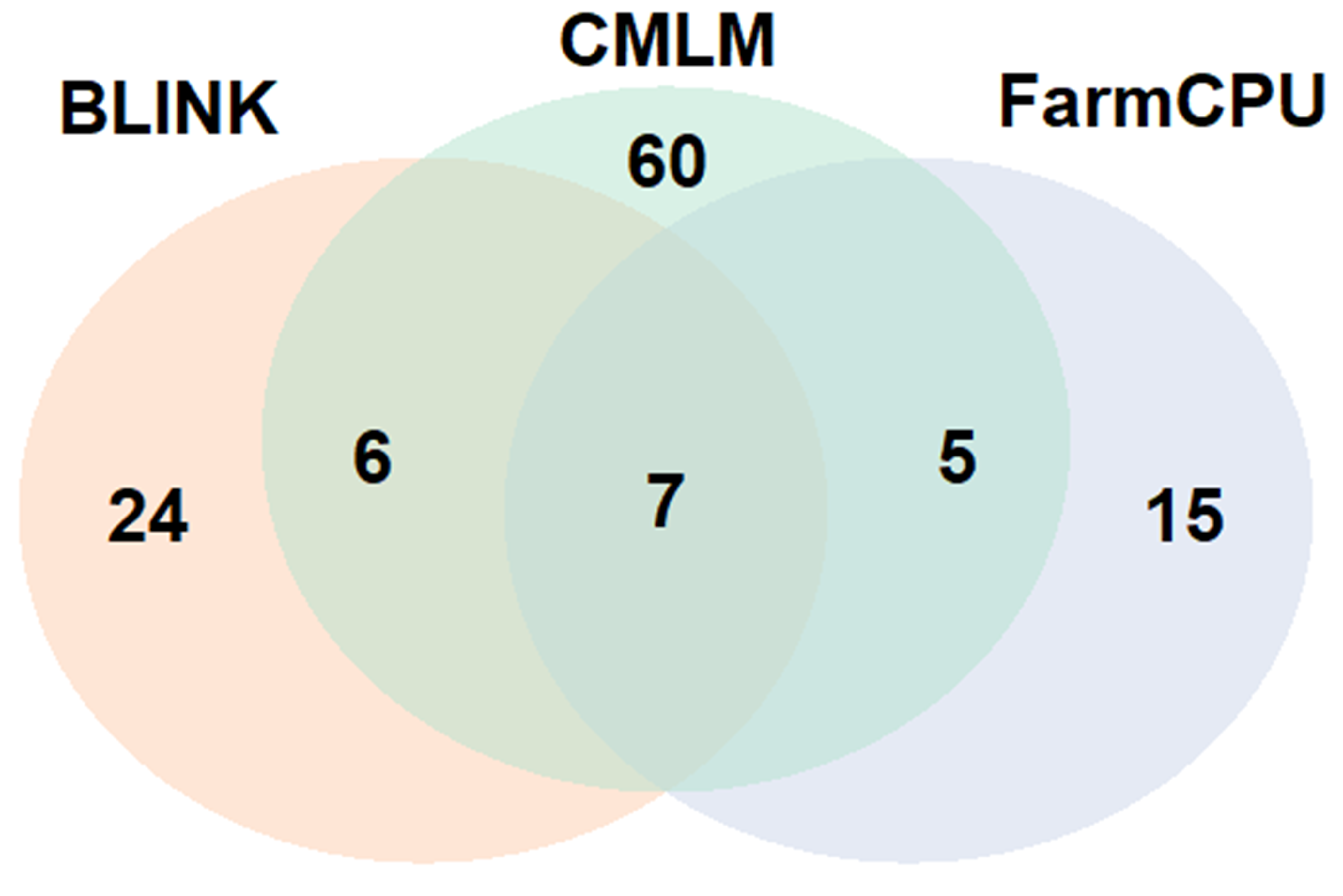

Supplement: Supplementary file 1 [file plants-13-03501-s001.zip › FIgure S4.tif]
